# Intakes of culinary herbs and spices from a food frequency questionnaire evaluated against 28-days estimated records

**DOI:** 10.1186/1475-2891-10-50

**Published:** 2011-05-16

**Authors:** Monica H Carlsen, Rune Blomhoff, Lene F Andersen

**Affiliations:** 1 Department of Nutrition, Institute for Basic Medical Sciences, University of Oslo, Norway

## Abstract

**Background:**

Worldwide, herbs and spices are much used food flavourings. However, little data exist regarding actual dietary intake of culinary herbs and spices. We developed a food frequency questionnaire (FFQ) for the assessment of habitual diet the preceding year, with focus on phytochemical rich food, including herbs and spices. The aim of the present study was to evaluate the intakes of herbs and spices from the FFQ with estimates of intake from another dietary assessment method. Thus we compared the intake estimates from the FFQ with 28 days of estimated records of herb and spice consumption as a reference method.

**Methods:**

The evaluation study was conducted among 146 free living adults, who filled in the FFQ and 2-4 weeks later carried out 28 days recording of herb and spice consumption. The FFQ included a section with questions about 27 individual culinary herbs and spices, while the records were open ended records for recording of herbs and spice consumption exclusively.

**Results:**

Our study showed that the FFQ obtained slightly higher estimates of total intake of herbs and spices than the total intake assessed by the Herbs and Spice Records (HSR). The correlation between the two assessment methods with regard to total intake was good (r = 0.5), and the cross-classification suggests that the FFQ may be used to classify subjects according to total herb and spice intake. For the 8 most frequently consumed individual herbs and spices, the FFQ obtained good estimates of median frequency of intake for 2 herbs/spices, while good estimates of portion sizes were obtained for 4 out of 8 herbs/spices.

**Conclusions:**

Our results suggested that the FFQ was able to give good estimates of frequency of intake and portion sizes on group level for several of the most frequently used herbs and spices. The FFQ was only able to fairly rank subjects according to frequency of intake of the 8 most frequently consumed herbs and spices. Other studies are warranted to further explore the intakes of culinary spices and herbs.

## Background

Humans have a long history of using herbs and spices in their daily life, as food flavouring, food preservation and for medicinal purposes [1,2]. The production and export of herbs and spices have increased worldwide the last two decades (http://faostat.fao.org/) and increasing use of herbs and spices in food flavouring is a global food trend [2]. Even so, little data exist regarding actual dietary intake levels of herbs and spices [2,3].

The leaf or herbaceous part of a plant, fresh or dried, used for flavouring in food preparation is often referred to as a culinary herb, whereas any other part of a plant, often dried, as a spice [1,2,4,5]. Examples of the latter are buds (cloves), bark (cinnamon/cassia), roots (ginger), berries (peppercorn) and aromatic seeds (cumin). In addition to pure spices, other food flavourings are mixed spice blends (henceforth called spices) and condiments (for example mustard paste). Herbs and spices are rich in phytochemical antioxidants [6]. Research indicates that herbs and spices, or their bioactive components, may act alone or in concert to reduce disease risk through their antimicrobial [4,5,7], antioxidant [8] and antitumorigenic properties [3,4]. These mechanisms of action are of particular interest when considering the role of oxidative stress and inflammation in chronic diseases such as cardiovascular disease and cancer.

Dietary intake assessments are in general complicated by measurement errors [9]. Estimating typical intake of herbs and spices is even more problematic because they are consumed in small amounts and often as integrated parts of prepared dishes. The concentrations of herbs and spices used in food preparation often falls within the range of 0.5 - 1.0% [7]. In addition, food and nutrient databases used in research for calculating food and nutrient intakes are only recently beginning to include data on herbs and spices [3].

The aim of our study was to perform an evaluation study, where the intakes of individual herbs and spices estimated from the food frequency questionnaire (FFQ) were compared to estimates from a reference method. The reference method was 28 days records of herb and spice consumption.

## Methods

### Subjects and study design

Details of the study design and enrolment of participants have been described previously [10]. After enrolment the participants received the FFQ and written instructions by mail and were asked to complete the FFQ at home. Within two weeks the participants attended a physical examination where the FFQ was returned and the participants were given written and oral instructions in how to perform the recording of herb and spice consumption. Data collection was carried out from September 2006 until March 2007. This study was conducted according to the guidelines laid down in the Declaration of Helsinki and all procedures involving human subjects were approved by the Regional Ethics Committee for Medical Research. Written informed consent was obtained from all subjects.

### The semi-quantitative FFQ

The questionnaire was designed to capture the habitual food intake the preceding year among Norwegian adults, with focus on food and beverages that are potential important sources of phytochemical antioxidants. Details of the FFQ have been described previously in Carlsen et al 2010 [10]. In short, the FFQ was designed to cover total energy intake of the population and included 250 questions about food items, grouped together according to the Norwegian food pattern. A section called "Spices" included questions about the intakes of individual herbs and spices. Based on sales information from two major manufacturers of culinary spices in Norway, questions about the consumption of 27 individual herbs and spices were included in the FFQ; dry and ground herbs and spices included were basil, black pepper, chili, cumin, turmeric, ginger, cardamom, cinnamon/cassia, curry spice blend, caraway, clove, oregano, paprika, piri piri, rosemary, sage, thyme, barbeque spice blend, and taco spice blend; fresh herbs and spices included were basil, chili, dill, ginger, oregano, peppermint, parsley and thyme. For those herbs and spices which commonly are used both fresh and dried (basil, chili, ginger, oregano and thyme) we asked about fresh and dried variants in separate questions. We asked for both frequencies of intake and portion sizes used. The options of frequency of consumption was 'never', 'less than once a month', '1', '2', '3', or '4 times per month', '2-4 times per week','5-7 times per week' and '8+ times per week'. The options for portion sizes were given in teaspoons, with 5 different values; 1/4, 1/2, 1, 2 and 3+ teaspoons. When a participant registered the frequency "never", he or she was defined as a non-consumer, otherwise the participant was defined as a consumer for that particular herb or spice. The FFQs were scanned and the image files transferred into data files using the Cardiff Teleform 2006 software. Frequencies and portion sizes were calculated using the food and nutrient database (KBS version 4.9, 2008, EA-07) developed at the Department of Nutrition, University of Oslo [11]. The food database KBS AE-07 is based on the 2006 edition of the Norwegian food composition table (http://old.matportalen.no/matvaretabellen/index_html/main_view_eng).

### The herbs and spice records

The 178 participants were supplied with a booklet and given written and oral instructions in how to record all consumption of culinary herbs and spices for 28 consecutive days. The herbs and spice record (HSR) was an open ended record were the participants recorded all use of herbs or spices every day. The consumption of herbs and spices was registered in teaspoons. Herbs and spices already added to premade food (meals in restaurants etc.) were not included in the recordings due to difficulties obtaining information about the amounts of herbs or spices in the pre-cooked food items. After completion the HSRs were returned to the University of Oslo by mail and checked for completeness, i.e. if registration of consumption was done for all 28 days. One hundred and forty-six participants returned complete HSRs. All intakes, frequencies of intake and portion sizes per eating occasion of herbs and spices from the HSR were computed using the food database AE-07 and KBS software system (KBS, version 4·9, 2008).

### Statistics

The intakes of individual herbs and spices are presented as frequency of intake in times per month and portion size in g per eating occasion. An 'eating occasion' was defined as any meal in witch the herb/spice in question was used, independent of day, during the time period covered by the registration method. All frequencies of intake and portion size data showed skewed distribution. However, median values of frequency and portion size were for most of the herbs and spices 0.01 or 0.00, which did not provide much useful information. We therefore chose to present both the mean and median values of frequency and portion sizes even though the median values are the more appropriate estimate for a skewed data set. The total intake of herbs and spices, that is, the sum of all herbs and spices per person is the only estimate given as g/person/day. Differences between the FFQ and the HSR in frequency of intake and portion size per eating occasion were tested with Wilcoxon signed-rank test. Correlations are Spearman Rank Order Correlation coefficients (r_s_) and defined as good for values of 0.5 and above, and fair for values between 0.3 and 0.5. In tables 1, 2 and 3 the participants were defined as consumers based on the intake data from the FFQ. Kappa statistic for the agreement between methods was performed, where a value of 0.21-0.40 is considered fair, 0.41-0.60 is considered moderate and 0.61-0.80 is considered as good agreement [12]. Results were considered to be statistically significant at p < 0.05. Data were analyzed using SPSS for Windows release 16.0 (SPSS Inc., Chicago, IL, USA).

**Table 1:** Mean and median frequencies of intake, in times per month, from the FFQ and the HSR respectively, and Spearman correlations between FFQ and HSR, for the 8 most frequently consumed herbs and spices in the study population.

	All participants n = 146					Consumers only					
		
	Mean		Median				Mean		Median		
							
	FFQ	HSR	FFQ	HSR	r_s_	n	FFQ	HSR	FFQ	HSR	r_s_
Basil, dry	2.0	0.8	0.01	0.00	0.3^a^	96	3.1	1.1	2.00	0.00	0.2
Basil, fresh	1.7	1.2	0.01	0.00	0.6 ^a^	87	2.4	2.0	1.00	1.00^b^	0.5 ^a^
Cinnamon/cassia	2.4	2.1	1.50	1.00 ^b^	0.5 ^a^	125	2.8	2.3	2.00	2.00 ^b^	0.5 ^a^
Oregano, dry	2.6	1.1	1.00	0.00	0.3 ^a^	114	3.4	1.3	2.00	0.00	0.3 ^a^
Pepper	14.7	9.8	12.00	10.00	0.4 ^a^	140	15.2	10.0	12.00	10.00	0.4 ^a^
Spice blend "Barbeque"	3.1	1.2	1.00	0.00	0.4 ^a^	103	4.0	1.5	2.00	0.00	0.4 ^a^
Spice blend "Curry"	1.3	0.9	0.01	0.00	0.3 ^a^	115	1.7	1.0	1.00	0.00	0.3 ^a^
Spice blend "Taco"	0.8	0.5	0.01	0.00	0.5 ^a^	89	1.4	0.7	1.00	0.00	0.4 ^a^

FFQ, food frequency questionnaire; HSR, herbs and spice record; r_s_, Spearman correlation coefficient.

^a ^correlation significant at 0.01 level

^b ^HSR frequency not significantly different from FFQ frequency, Wilcoxon signed-rank test

Data shown for all participants and consumers only.

**Table 2:** Mean and median portion sizes per eating occasion, in g per eating occasion, from the FFQ and the HSR respectively, and Spearman correlations between FFQ and HSR, for the 8 most frequently consumed herbs and spices in the study population.

	All participants n = 146					Consumers only					
		
	Mean		Median				Mean		Median		
							
	FFQ	HSR	FFQ	HSR	r_s_	n	FFQ	HSR	FFQ	HSR	r_s_
Basil, dry	0.5	0.2	0.3	0.0	0.3^a^	96	0.7	0.3	0.5	0.0	-
Basil, fresh	0.7	0.9	0.5	0.0^b^	0.5 ^a^	87	1.1	1.5	1.0	0.6 ^b^	0.3 ^a^
Cinnamon/cassia	1.1	1.9	0.7	0.8 ^b^	0.5 ^a^	125	1.3	2.0	1.3	1.0 ^b^	0.1
Oregano, dry	0.3	0.1	0.2	0.0	0.3 ^a^	114	0.3	0.2	0.2	0.0	0.3 ^a^
Pepper	1.3	1.2	0.6	1.0 ^b^	0.3 ^a^	140	1.3	1.2	0.6	1.0 ^b^	0.3 ^a^
Spice blend "Barbeque"	1.2	1.5	0.8	0.0 ^b^	0.3 ^a^	103	1.6	1.8	1.5	0.0 ^b^	0.1
Spice blend "Curry"	1.3	0.9	0.9	0.0	0.2 ^a^	115	1.6	1.0	1.1	0.0	0.3 ^a^
Spice blend "Taco"	1.2	2.1	0.6	0.0 ^b^	0.5 ^a^	89	1.9	3.0	1.3	0.0 ^b^	0.3 ^a^

FFQ, food frequency questionnaire; HSR, herbs and spice record; r_s_, Spearman correlation coefficient.

^a ^correlation significant at 0.01 level

^b ^HSR portion size not significantly different from FFQ portion size, Wilcoxon signed-rank test

Data shown for all participants and consumers only.

**Table 3:** Cross classification of consumers according to frequency of intake and kappa coefficients for the 8 herbs and spices most frequently consumed in the study population

	**n**	**% correct quartile**	**% adjacent quartile**	**% opposite quartile**	**kappa**
	
Basil, dry	96	42	43	2	0.23
Basil, fresh	87	63	29	0	0.51
Cinnamon/cassia	125	51	40	0	0.35
Oregano, dry	114	52	42	2	0.36
Pepper	140	41	48	3	0.21
Spice blend, Barbeque	103	55	36	1	0.40
Spice blend, Curry	115	62	31	0	0.49
Spice blend, Taco	89	61	36	0	0.48

FFQ, food frequency questionnaire; HSR, Herbs and spice record.

Consumers defined by the FFQ.

## Results

Our study population consisted of 146 participants (63 men and 83 women), with mean age 47 (95% CI 44, 49) years and mean BMI 25.0 (95% CI 24.4, 25.7) kg/m^2^. Fourteen women and 11 men were current smokers. Age and number of current smokers did not differ significantly between the genders.

**Figure 1:**
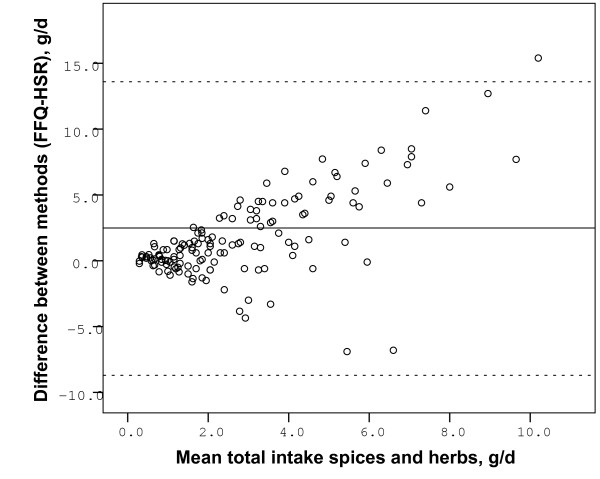
**Bland-Altman plot of total intake of herbs and spices**. Bland-Altman plot of the difference between herb and spice intake from the FFQ and the HSR, against the mean of the FFQ and HSR intakes, for each participant. The solid line represents the average difference between the intakes from the FFQ and the HSR (2.5 g/d for the whole study population, 1.7 g/d without 4 extreme outliers). The dotted lines are upper and lower limits of agreement (mean ± 1.96 SD) in this population. 4 extreme outliers of intake from the FFQ are excluded form the plot but not from the calculations.

Median estimates of total herb and spice consumption in our study population was 2.7 g/person/day (range 0.19 to 45.0, interquartile range 4.4) from the FFQ and 1.6 g/person/day (range 0.00 to 10.0, interquartile range 1.8) from the HSR. The estimate from the FFQ was significantly higher than the estimate from the HSR (p < 0.01). There was no significant difference in total intake of herbs and spices between men and women, estimated by the FFQ (p = 0.9) and by the HSR (p = 0.5). The correlation between the FFQ and HSR estimates of total intake of herbs and spices was 0.5 for all participants, 0.4 for women and 0.6 for men (p < 0.01). The Bland Altman plot of total herb and spice intake (Figure 1) shows that the differences in intake between the two methods (FFQ-HSR) increase with higher mean values of intake. Moreover the plot shows a tendency of overestimation of intake by the FFQ. Mean difference in total intake between FFQ and HSR was 2.5 g/d. When we exclude 4 extreme outliers with intakes from the FFQ above 20 g/d, the mean difference in total intake was 1.7 g/d.

Of the 27 different herbs and spices investigated only 8 were consumed by 1/3 or more of the study population. In the following evaluation these 8 most consumed herbs and spices were analysed. The intakes of individual herbs and spices in our study were very low, resulting in intake values lower than 0.01 g/person/day for many of the participants and for the mean and median intakes of the majority of herbs and spices. Therefore, the intakes of the individual herbs and spices are presented as frequency of intake in times per month, and portion size per eating occasion in g, and not in g/person/day.

Table 1 presents the mean and median frequencies of intake of the 8 herbs and spices, assessed by the FFQ and the HSR, respectively. The frequency estimates are presented for the whole study populations as well as for the consumers only. In the whole study population mean frequencies of intake estimated from the FFQ were significantly higher for 7 of the 8 herbs and spices when compared to the HSR. The frequency of intake of cinnamon/cassia was not significantly different between the methods (Table 1). The Spearman correlation coefficients between frequencies of intake were fair (0.3 or 0.4) for dry basil, dry oregano, pepper, barbeque spice blend, and curry spice blend. For fresh basil, cinnamon/cassia and taco spice blend, the correlation coefficients were good (0.5 to 0.6).

When evaluating the frequencies of intake for the consumers only we observed approximately similar results as for the whole study population (Table 1). However, the frequencies of fresh basil, as well as the frequency for cinnamon/cassia, were now not significantly different between methods. The correlation coefficients of frequencies for consumers were almost similar to those for the whole study population (Table 1).

Table 2 presents the mean and median portion sizes per eating occasion for the 8 herbs and spices, assessed by the FFQ and the HSR respectively. When evaluating the whole study population, no significant difference in portion sizes per eating occasion were observed when comparing estimates from the FFQ with estimates from the HSR, for fresh basil, black pepper, barbeque spice blend and taco spice blend. The portion sizes for dry basil, dry oregano and curry spice blend were significantly higher compared to the HSR. The portion size of cinnamon/cassia was lower as compared to the HSR (Table 2). The correlation coefficients observed in the whole study population were fair (0.3 or 0.4) for dry basil, dry oregano, pepper and taco spice blend, and good (0.5) for fresh basil, cinnamon/cassia and taco spice blend (Table 2). Curry spice blend showed low correlation between portion estimates from the HSR and the FFQ.

When we evaluated the portion sizes for consumers only, approximately the same patterns were observed as for the whole study population. However, now the portion size for cinnamon/cassia was not significantly different between the methods, thus 5 of 8 portion sizes from the FFQ were similar to the corresponding portion sizes from the HSR. The correlation coefficients for the consumers were generally lower than for the whole study population (Table 2).

Cross-classification of consumers into quartiles of frequencies of intake, and Kappa coefficients of agreement, for the 8 most frequently consumed herbs and spices, is presented in Table 3. The non-consumers were excluded from the calculations because if they were included, classification would not be possible due to a large number of null frequencies. Only a small percentage of the consumers were grossly misclassified and between 85 and 97% of the consumers were classified into correct or adjacent quartile (Table 3). Cross-classification of total intake of herbs and spices (in g/person/day, n = 146) showed that 40% of the participants were classified into correct quartile, 41% were classified into adjacent quartile of frequency, whereas 3% were grossly misclassified according to total herbs and spice intake.

The frequencies of intake and portion sizes for the other herbs and spices included in the FFQ are provided in Additional file 1, Table S1.

## Discussion

In 2003 Sasaki et al. [13] presented an evaluation of total intake of seasoning and spice from a FFQ used in the Japan Public Health Center based prospective study on cancer and cardiovascular diseases. The food intakes from the Japanese FFQ were evaluated against 28-d and 14-d dietary records and the study showed that the FFQ underestimated the total intake of seasoning and spice (average intake 5 g/d) by more than 85% [13]. In 2007 Pellegrini et al. [14] evaluated a FFQ developed to assess total antioxidant capacity in the Italian diet. Included in the evaluation was a comparison of the intake of total spices from the FFQ and from a 3-d weighed food record. The results showed that the FFQ overestimated total intake of spices by on average 3.2 g per day. In the present study the FFQ overestimated total intake of herbs and spices by on average 1.1 g per day. In the Japanese and Italian studies only total spice consumption was evaluated. Our present study is to our best knowledge the first study that has assessed and evaluated the intakes of individual herbs and spices.

The assessment of herb and spice consumption in a whole population as well as among consumers only may be of interest. Therefore, the data have been analysed in both groups. For many of the herbs and spices investigated in our study, the number of non-consumers was high, thus we were forced to focus our method evaluation on the herbs and spices that were most frequently consumed.

In our evaluation we observed a general trend of overestimation of frequency of use, among consumers as well as in the whole study population. Only cinnamon/cassia showed similar frequencies using the two assessment methods in the whole study population. The frequencies from the FFQ that differed from the HSR were overestimated by approximately 1 to 2 times per month, except for dried black pepper for which the difference was approximately 5 times per month.

When evaluating the consumers only, the frequencies of intake from both methods increased slightly as compared to the results for the whole study population. For fresh basil and cinnamon/cassia the correlation coefficients were good. Thus, for fresh basil and cinnamon/cassia the FFQ achieved good estimates of frequency of intake compared to the HSR, in addition to good correlation between the frequency estimates from the two methods, in both the whole study population and among consumers only. Moreover, the classification into quartiles of frequency suggests that the FFQ may be used to classify and rank participants according to relative frequency of intake of fresh basil and cinnamon/cassia.

The evaluation of portion sizes showed that, in the whole study population, the differences in portion size per eating occasion from the FFQ were not significantly different from the HSR portion sizes for 4 of the herbs and spices investigated, whereas 3 were less than 0.4g higher than those from the HSR. This pattern of estimates was also observed for consumers only. This suggests that the present FFQ may obtain estimates of portion size per eating occasion that are good estimates on group level for the most frequently consumed herbs and spices. However, the correlation coefficients obtained for portion sizes showed more variation and lower values than the correlation for frequencies of intake.

Based on the numbers of participants correctly classified and the associated Kappa values, we find that the FFQ has moderate ability to classify participants into quartiles of frequency of intake.

Our study participants were limited to estimate and record the consumption of herbs and spices that they used when they prepared and consumed a meal. Herbs and spices in premade food, such as meals in restaurants and precooked take-away meals were not recorded in either of the methods. Depending on the nature of the meals, these excluded meals may be important sources of herbs and spices, thus the total intake of herbs and spices in our study may be underestimated.

Another limitation of this study is the discrepancy of time periods covered by the FFQ and the HSR, the preceding year and 28 days, respectively. A reference method which covered one year is preferable and may have reduced the differences between estimates from the FFQ and the HSR.

## Conclusions

In summary, our evaluation study showed that it is possible to obtain fair estimates of total intake of herbs and spices using the FFQ. The correlation between the two assessment methods with regard to total intake was good, and the cross-classification suggests that the FFQ may be used to classify subjects according to total herb and spice intake. With regard to intakes of 8 individual herbs and spices, the FFQ obtained good estimates for median frequency of intake of 2 herbs/spices, while good estimates for portion sizes were obtained for 4 out of 8 herbs/spices in the whole study population and 5 out of 8 among the consumers only. Portion sizes showed higher agreement among consumers than in the whole study population. The correlations between frequencies of intake estimated by the FFQ and the HSR, as well as the cross-classification suggested that the FFQ was able to fairly rank subject according to frequency of intake of the 8 most frequently consumed herbs and spices.

## List of abbreviations

FFQ: Food Frequency Questionnaire; HSR: Herb and Spice Record.

## Competing interests

The authors declare that they have no competing interests.

## Authors' contributions

MHC was responsible for study design, design of the FFQ and the HSR, participant recruitment, data collection, statistical analyses and preparation of manuscript. RB was responsible for funding, and contributed to study design and manuscript revision, and LFA contributed to study design, design of the FFQ and the HSR, statistical analyses and manuscript revision. All authors read and approved the final manuscript.

## Supplementary Material

Additional file 1 **Table S1. Frequencies of intake and portions sizes for herbs and spices assessed in our study population**. the Additional file 1 contains Table S1 that presents the intake data of the additional spices and herbs investigated in our study, which are not presented in the main manuscript. Click here for file

## References

[B1] Davidson A The Oxford Companion to Food 2010 Oxford: Oxford University Press

[B2] Tapsell LC Hemphill I Cobiac L Patch CS Sullivan DR Fenech M Roodenrys S Keogh JB Clifton PM Williams PG Fazio VA Inge KE Health benefits of herbs and spices: the past, the present, the future Med.J.Aust 185 4 Suppl S4 24 21-8-2006 10.5694/j.1326-5377.2006.tb00548.x 17022438

[B3] Kaefer CM Milner JA The role of herbs and spices in cancer prevention J Nutr Biochem 2008 19 347 361 10.1016/j.jnutbio.2007.11.003 18499033 PMC2771684

[B4] Lai PK Roy J Antimicrobial and chemopreventive properties of herbs and spices Curr Med Chem 2004 11 1451 1460 15180577 10.2174/0929867043365107

[B5] Suppakul P Miltz J Sonneveld K Bigger SW Antimicrobial properties of basil and its possible application in food packaging J Agric Food Chem 2003 51 3197 3207 10.1021/jf021038t 12744643

[B6] Carlsen MH Halvorsen BL Holte K Bohn SK Dragland S Sampson L Willey C Senoo H Umezono Y Sanada C Barikmo IE Berhe N Willett WC Phillips KM Jacobs DR Jr Blomhoff R The total antioxidant content of more than 3100 foods, beverages, spices, herbs and supplements used worldwide Nutr J 2010 9 3 10.1186/1475-2891-9-3 20096093 PMC2841576

[B7] Shelef LA Antimicrobial effects of spices Journal of Food Safety 1984 6 29 44 10.1111/j.1745-4565.1984.tb00477.x

[B8] Zheng W Wang SY Antioxidant activity and phenolic compounds in selected herbs J Agric Food Chem 2001 49 5165 5170 10.1021/jf010697n 11714298

[B9] Willett W Nutritional Epidemiology 1998 Oxford: Oxford University Press

[B10] Carlsen MH Lillegaard IT Karlsen A Blomhoff R Drevon CA Andersen LF Evaluation of energy and dietary intake estimates from a food frequency questionnaire using independent expenditure measurement and weighed food records Nutr J 2010 9 37 10.1186/1475-2891-9-37 20843361 PMC2949781

[B11] Rimestad AH Løken EB Nordbotten A The Norwegian food composition table and the database for nutrient calculations at the Institute of Nutrition Reserach Nor J Epidemiol 2000 10 7 16

[B12] Bowers D Medical Statistics from Scratch 2008 Chichester: John Wiley & Sons Ltd. England

[B13] Sasaki S Kobayashi M Tsugane S Validity of s self-administered food frequency questionnaire used in the 5-year follow-up survey of teh JPHC study cohort I: comparison with dietary records for food groups Journal of Epidemiology 2003 13 s57 s63 12701632 10.2188/jea.13.1sup_57 PMC9767694

[B14] Pellegrini N Salvatore S Valtuena S Bedogni G Porrini M Pala V Del RD Sieri S Miglio C Krogh V Zavaroni I Brighenti F Development and validation of a food frequency questionnaire for the assessment of dietary total antioxidant capacity J Nutr 2007 137 93 98 17182807 10.1093/jn/137.1.93

