# Supplementary material for: Intakes of culinary herbs and spices from a food frequency questionnaire evaluated against 28-days estimated records
Source: Nutr J. 2011 May 16;10:50. doi: 10.1186/1475-2891-10-50 (PMC3116467; doi:10.1186/1475-2891-10-50)
Supplement: Additional file 1 — Table S1. Frequencies of intake and portions sizes for herbs and spices assessed in our study population. the Additional file 1 contains Table S1 that presents the intake data of the additional spices and herbs investigated in our study, which are not presented in the main manuscript. [file 1475-2891-10-50-S1.DOC]

Additional file 1, for manuscript: Intakes of culinary herbs and spices from a food frequency questionnaire evaluated against 28-days estimated records. Monica H Carlsen, Rune Blomhoff and Lene F Andersen

Table S1. Frequencies of intake and portions sizes for herbs and spices assessed in our study population.

|  | Frequency of intake, times/month | | | | |  | Portion size in mg / eating occasion | | | | |
| --- | --- | --- | --- | --- | --- | --- | --- | --- | --- | --- | --- |
|  | FFQ | |  | HSR | |  | FFQ | |  | HSR | |
|  | mean | median |  | mean | median |  | mean | median |  | mean | median |
| Caraway,dry | 0.26 | 0.00 |  | 0.18 | 0.00 |  | 391 | 0 |  | 110 | 0 |
| Cardamom, dry | 0.81 | 0.01 |  | 0.50 | 0.00 |  | 686 | 500 |  | 120 | 0 |
| Chili peppers,capsicum, dry | 1.23 | 0.01 |  | 0.73 | 0.00 |  | 598 | 600 |  | 300 | 0 |
| Clove, dry | 0.31 | 0.00 |  | 0.12 | 0.00 |  | 270 | 0 |  | 40 | 0 |
| Cumin, dry | 0.33 | 0.00 |  | 0.00 | 0.00 |  | 203 | 0 |  | 0 | 0 |
| Dill, fresh | 0.80 | 0.01 |  | 0.22 | 0.00 |  | 266 | 300 |  | 120 | 0 |
| Ginger, dry | 0.54 | 0.00 |  | 0.47 | 0.00 |  | 422 | 0 |  | 140 | 0 |
| Ginger, fresh | 1.38 | 0.00 |  | 0.72 | 0.00 |  | 1275 | 0 |  | 790 | 0 |
| Minth, fresh | 0.54 | 0.00 |  | 0.10 | 0.00 |  | 88 | 0 |  | 50 | 0 |
| Oregano, fresh | 0.53 | 0.00 |  | 0.08 | 0.00 |  | 140 | 0 |  | 50 | 0 |
| Parsley, fresh | 2.17 | 0.01 |  | 1.03 | 0.00 |  | 431 | 300 |  | 360 | 0 |
| Piri piri peppers, capsicum, dry | 0.12 | 0.00 |  | 0.00 | 0.00 |  | 115 | 0 |  | 0 | 0 |
| Rosemary, dry | 1.03 | 0.01 |  | 0.40 | 0.00 |  | 455 | 400 |  | 180 | 0 |
| Sage, dry | 0.19 | 0.00 |  | 0.05 | 0.00 |  | 103 | 0 |  | 10 | 0 |
| Sweet pepper, capsicum, dry | 1.48 | 0.01 |  | 0.69 | 0.00 |  | 799 | 500 |  | 590 | 0 |
| Thyme, dry | 1.53 | 0.01 |  | 0.53 | 0.00 |  | 266 | 200 |  | 130 | 0 |
| Thyme, fresh | 0.81 | 0.00 |  | 0.24 | 0.00 |  | 221 | 0 |  | 180 | 0 |
| Turmeric, dry | 0.21 | 0.00 |  | 0.08 | 0.00 |  | 342 | 0 |  | 40 | 0 |
